# Role of ellagic acid in regulation of apoptosis by modulating novel and atypical PKC in lymphoma bearing mice

**DOI:** 10.1186/s12906-015-0810-5

**Published:** 2015-08-15

**Authors:** Sudha Mishra, Manjula Vinayak

**Affiliations:** Biochemistry & Molecular Biology Laboratory, Centre of Advanced Study in Zoology, Banaras Hindu University, Varanasi, 221005 India

**Keywords:** PKC isozymes, Apoptosis, LDH-A, Anaerobic glycolysis

## Abstract

**Background:**

Protein kinase C regulates various cellular processes including cell proliferation, cell adhesion, apoptosis, angiogenesis, invasion, and metastasis. Activation of different PKC isozymes results in distinct cellular responses. Novel PKCs are mainly involved in apoptotic process. Atypical PKC subfamily plays a critical role in cell proliferation and apoptosis, cell differentiation and motility. However, Atypical PKCs show contradictory regulation in different tissues or cancer cells. The mechanism of diversified effects is not well explored. Antioxidant ellagic acid shows hepatoprotective, anti-carcinogenic and anti-mutagenic properties. Present study is focused to analyze the effect of ellagic acid on novel and atypical isozymes of PKC in regulation of PKC-mediated apoptosis in liver of lymphoma bearing mice. Implication of ellagic acid treatment to DL mice was analyzed on caspase-3 mediated apoptosis via PKCδ induced activation; and on maintenance of adequate supply of energy during cancer growth.

**Methods:**

15–20 weeks old adult DL mice were divided into four groups (*n* = 6). Group 2, 3, 4 were treated with different doses of ellagic acid (40 mg/kg, 60 mg/kg and 80 mg/kg bw). The mice were sacrificed after 19 days of treatment and liver was used for study. The effect of ellagic acid was determined on expression of novel and atypical PKC isozymes. Apoptotic potentiality of ellagic acid was checked on activities of caspase-3 and PKCδ in terms of their catalytic fragments. Aerobic glycolysis was monitored by LDH activity, especially activity of LDH A.

**Results:**

Ellagic acid treatment caused up regulation of expression of almost all novel and atypical PKC isozymes. Activities of PKCδ and caspase-3 were enhanced by ellagic acid, however activities of total LDH and LDH-A were inhibited.

**Conclusion:**

The results show that ellagic acid promotes apoptosis in lymphoma bearing mice via novel and atypical PKCs which involves PKCδ induced caspase-3 activation; and inhibition of glycolytic pathway.

## Background

Different isozymes of PKC are implicated in regulation of cellular processes including cell proliferation, differentiation, malignant transformation and apoptosis [1–3]. Members of PKC family catalyze either pro- or anti-apoptotic processes depending on the isoforms and cellular context. Previously we have reported over-expression of classical PKCs in lymphoma bearing mice which is known to favour cell proliferation [4]. Novel PKCs are mainly involved in apoptotic process [5]. PKCδ deficient mouse is reported to suppress apoptosis in response to chemotherapeutic drug [6]. Ionising radiation induces apoptosis via activation of PKC*δ* in Jurkat T cells [7]. Increased expression and/or activation of PKCή has been associated with cell cycle arrest in a various kinds of cell lines as well as in a variety of normal epithelial and non epithelial tissues [8]. Atypical Isozymes of PKC subfamily including PKCζ and PKCι, are involved in different cellular processes like cell proliferation, apoptosis and differentiation. They are found either up regulated or down regulated in different cancers. PKCζ is reported to be involved in suppressing melanoma cell migration [9]. However, PKCι can function as an oncogene [10]. Studies on PKCι have revealed a critical role of PKCι at multiple stages of tumorigenesis, including tumor initiation, progression, and metastasis. However, little is known about its role in apoptosis.

Murine Dalton’s lymphoma is a Non Hodgkin’s transplantable T cell lymphoma which can spread beyond the lymphatic system to almost any part of the body including liver, bone marrow and spleen. Secondary lymphoma growth in liver has been confirmed earlier in our laboratory by histopathological analysis showing impaired shape of hepatocytes with large nucleus, increased sinusoids with infiltration of a large number of leucocytes [11]. Liver of DL mice was found larger and softer as compared to normal ones indicating malignant growth.

Apoptosis or programmed cell death is a physiological process through which cell population regulates normal growth and morphogenesis. Alterations of various signaling pathways may result in deregulation of apoptosis, leading to cancer. Continuous increase in cell population is characteristic feature of any cancer which may occur due to defect in cell cycle controlling mechanism or in any apoptotic pathway. Caspase-3 is a member of the caspase family that plays a central role in the execution of apoptotic program. The enzyme is involved in both extrinsic as well as extrinsic pathways of apoptosis, targeting various mediators as confirmed by both *in vitro* and *in vivo* studies [12]. Caspase-3 is activated only in the form of cleaved p17 peptide. PKCδ phosphorylates and cleaves caspace-3 to its active fragment i.e. p17 peptide and is itself a target for caspases. Caspase-3 targets the cleavage of PKCδ resulting into its constitutively active catalytic fragment (CF) which is involved in apoptosis [13, 14].

High energy requirement for cancer growth is met with aerobic glycolytic metabolism. Alternation in cellular metabolism to anaerobic state is most consistent hallmark of cancer [15]. LDH-A is the main isozyme of glycolytic enzyme LDH, kinetically favouring conversion of pyruvate to lactate under aerobic and hypoxic conditions. Decreased activity of LDH-A is reported to reduce cellular transformation, and markedly delayed tumor formation indicating that LDH-A is important for tumor initiation [16]. Therefore decreased activity of LDH is expected to suppress survival of cancerous cells by depletion of high energy requirement.

The link between different phytochemicals and their cancer preventive role has been widely investigated. Ellagic acid, a polyphenolic compound found in berries, fruits and nuts is reported to possess growth-inhibiting and apoptotic activities in different cancer cell lines *in vitro* [17, 18]. We have earlier reported suppression of lymphoma growth in mice by ellagic acid [4, 19]. The present study is aimed to investigate molecular signaling pathway involved in anti carcinogenic activity of ellagic acid via modulation of apoptotic process and its correlation with novel and atypical isozymes of PKC. Further, impact of ellagic acid on glycolytic metabolism is analyzed in liver of lymphoma bearing mice.

## Methods

All the chemicals used in experimental work were of analytical and molecular biology grade. TEMED, 2-thiobarbituric acid, ellagic acid and SDS were purchased from Himedia. Reagents for RNA isolation, RT-PCR, antibody of β-actin, PKC δ, caspase-3 and PMSF were purchased from Sigma Aldrich, Ribonuclease inhibitor, random hexamer, 100 bp Plus DNA ladder; and Reverse Transcriptase from Fermentas Life sciences, Polyclonal HRP-conjugated goat anti-rabbit IgG from Bangalore Genei, ECL super signal Western pico kit from Pierce respectively.

### Induction of lymphoma

AKR strain mice were used for the study as they are much more susceptible to lymphoma and have a short life span (approximately 18 months). The study was approved by Animal Ethical Committee of Banaras Hindu University.  Mice were bred and maintained as per the norms of Animal Ethical Committee of Institute, at 25 ± 2 °C under 12 hrs light/dark schedule with ad libitum supply of standard mice feed and drinking water.Dalton’s lymphoma ascites cells were transplanted in adult male mice through serial transplantation by intra peritoneal (i.p.) injection of about 1 × 10^6^ viable ascites tumour cells in 1 ml of phosphate buffer saline (PBS) per mouse, as described previously [19]. Development of DL was confirmed by abdominal swelling and increased body weight, which were visible clearly on 10–11 day post transplantation.

### Administration of ellagic acid

DL mice were divided into four groups (*n* = 6). Three groups were treated with different doses of ellagic acid (40 mg/kg, 60 mg/kg and 80 mg/kg body weight) dissolved in 0.2 % DMSO as a vehicle. Treatment was started from the next day of ascites cell transplant. Ellagic acid was given by gavage feeding at 24 h intervals for 15 consecutive days. One DL control group was given only vehicle. All the groups of mice were sacrificed on day 19 of DL transplantation by cervical dislocation. Liver was excised immediately after sacrificing the animal and washed in chilled normal saline. Liver of all animals of one group was pooled and mixed by chopping aseptically at 4 °C for average result. Collected tissue was used immediately or preserved at −80 °C for further study.

### Assay of Lactate Dehydrogenase (LDH)

Assay of total LDH was performed in liver of mice according to the method of Schwartz and Bodansky [20] with minor modifications [21]. The principle of detection is based on spectrophotometrically monitored conversion of NADH to NAD+ in a pyruvate utilizing reaction at 340 nm.

Activity of LDH-A was analyzed by activity gel assay. Proteins were separated on 7.5 % resolving gel containing 4.5 % stacking gel using Tris/ glycine, pH 8.3 electrophoresis buffer; and stained by activity staining with solution containing 0.25 M Phosphate buffer (pH 7.4), 10 mM lithium lactate, 15 mM NAD+, 1.25 mM NBT, 5 mM NaCN, 5 mM MgCl2, 1.25 mM, and 3.3 mM phenazine methosulfate in dark for 30–45 min at RT. The reaction was stopped after appearance of desired bands by adding fixative solution (45 % methanol and 10 % acetic acid). The level of LDH-A was analyzed by densitometry scanning of appeared band on gel using Alpha image analyzer software.

### RNA Isolation and RT-PCR

Expression of different genes was studied by semiquantitative RT-PCR. Total RNA from each group of tissue was isolated separately using TRI reagent (Sigma Aldrich, Catalogue Number-T9424) as per the manufacturer’s instruction.

DNase treatment was carried out on total RNA using TURBO DNA-Free™ Kit I (DNase I, Ambion). Reverse transcription of 2 μg RNA was done by using c-DNA synthesis kit (Revert Aid^TM^ First Strand c-DNA synthesis Kit, Fermentas Life Science, Catalogue Number-K1621), according to manufacturer’s standard protocol. cDNA was amplified using specific primers supplied by Metabion International AG (Table 1). Amplification was performed in a DNA Thermal Cycler (Applied Biosystem) under a three-step temperature cycle. The band intensity of amplified products in the gel was visualized, photographed and analyzed using Gel Doc System (Alpha InnotechEC). Further, band intensity was normalized with β-actin.

**Table 1:** Primer pairs and conditions of PCR

Genes with accession no	Sequences of primer pairs (F: forward, R: reverse)	PCR condition (annealing)	No. of cycles	Amplicon Size (bp)
PKCδ	F: 5′-AGGCCGTGTTATCCAGATTG-3′	58 °C-40 s	27	426
(NM_011103.3)	R: 5′-CGGCCGATAATCTTGTCAAT-3′			
PKCη	F: 5′-CAAACTGCGGGGTGAATG-3′	54 °C-30 s	27	437
(NM_008856.3)	R: 5′-TCCGTTCACAAACTCCATGA-3′			
PKCε	F: 5′-ACGAGTGTTCAGGGAGCGTA-3′	58 °C-45 s	27	331
(NM_011104.3)	R: 5′-CGTGGGGACCTTGTAGTTGT-3′			
PKCζ	F: 5′-TCAAGTGGGTGGACAGTGAA-3′	57 °C-45 s	30	284
(NM_008860.3)	R: 5′-CCATATCCTTTCGCTGCACT-3′			
PKCι	F: 5′-GGGACTTTGCAGTGAGGTTC-3′	56 °C-40 s	28	192
(NM_008857.3)	R: 5′-CGCTCTGGTACACATGGAAA-3′			
PKCθ	F- 5′-CCAGAAAAAGCCAACCATGT-3′	57 °C-30 s	35	521
(NM_008859.2)	R- 5′-TGGTTTCTCGGCTATTGATTG-3′			
Caspase-3	F- 5′- CAAAGCGCAGTGTCCTGCGG-3′	61 °C-45 s	28	546
(NM_009810.3)	R- 5′- ACCCCGGCAGGCCTGAATGA-3′			
LDH-A	F- 5′-ATGCACCCGCCTAAGGTTCTT-3′	55 °C-30 s	28	103
(NM_010699.2)	R- 5′-TGCCTACGAGGTGATCAAGCT-3′			
β-actin	F: 5-GTGGGCCGCCCTAGGCACCAG-3′	56 °C-45 s	27	539
(NM_007393.3)	R: 5-TCTTTGATGTCACGCACGATTTC-3′			

### Western blot analysis

Catalytic activity of PKCδ and caspase-3 were determined by Western blot analysis. Equal amount of protein from each sample was separated in 10 % SDS–PAGE and transferred to the PVDF membrane overnight at 4 °C. Membrane was blocked in 5 % non-fat milk in PBS (pH 7.4) for 2 h ar RT. The blot was then incubated with rabbit anti- caspase-3(Product number C8487) or rabbit anti- PKCδ (AB-645; Product number SAB-4300539) in 1 % BSA and 0.05 % Tween-20 in PBS (pH 7.4). Blot was washed and incubated with HRP-conjugated goat anti-rabbit IgG in PBS (pH 7.4) containing 1 % BSA and 0.05 % Tween-20 for 2 h at RT. Immunoreactive proteins were detected with ECL super signal kit (Pierce Biotechnology) in X-ray film. The same blot was used for the detection of β-actin as internal control. Band density values were normalized with β-actin.

### Statistical analysis

All experiments were repeated three times and statistical analysis was performed using SPSS version 11.5 software with one-way analysis of variance (ANOVA) followed by Tukey test. Values are expressed as mean ± SD. A value *p* < 0.05 was taken as statistically significant from DL.

## Results

### Ellagic acid up regulates the expression of novel PKC isozymes

**Fig. 1:**
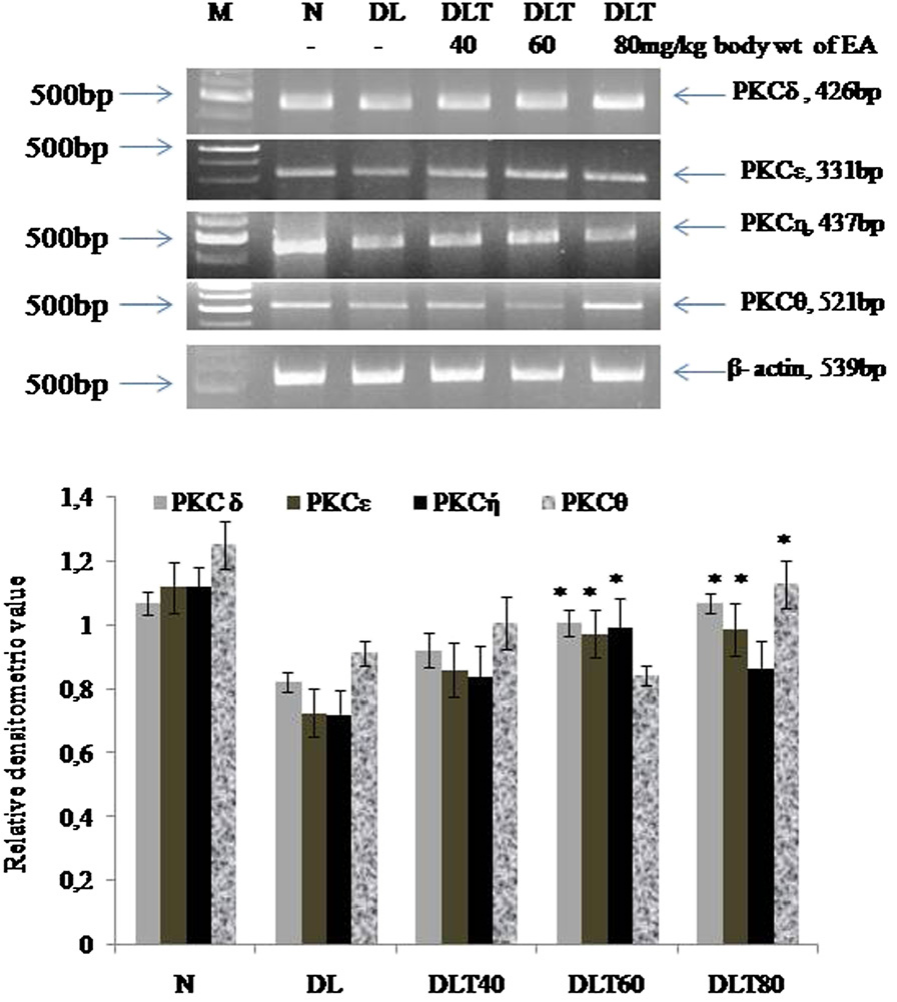
Effect of ellagic acid on gene expression of novel PKC isozymes in liver of DL mice Values are expressed as mean ± SD, * indicates that groups differ significantly from DL at the level of significance *p < 0.05* using one way ANOVA followed by Tukey test. N, DL, DLT40, DLT60 and DLT80 represents normal, Dalton’s lymphoma bearing, and Dalton’s lymphoma bearing mice treated with 40, 60, 80 mg/kg body weight of ellagic acid respectively. EA – Ellagic acid, M- Marker

The expressions of all four novel isoforms were down regulated in DL mice liver as compared to normal. However, ellagic acid treatment to DL mice was found to cause up regulation in mRNA transcription of all isozymes as compared to untreated DL mice. Up regulation in expression of PKCδ, PKCε, PKCή was observed approximately up to 1.2 folds, 1.34 folds and 1.37 folds respectively with a dose of 60 mg ellagic acid. However, 80 mg dose increased the expression of all novel PKC isozymes (δ, ε, ή and θ) upto 1.3 folds, 1.36 folds, 1.3 folds and 1.24 folds respectively (Fig. 1).

### Ellagic acid up regulates activity of PKCδ

**Fig. 2:**
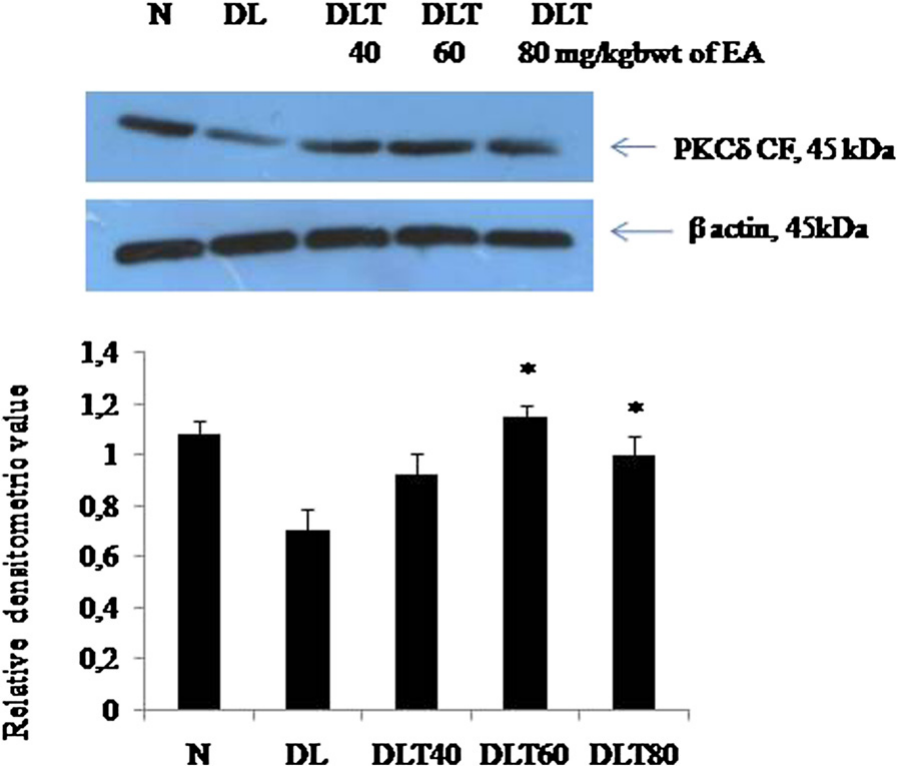
Effect of ellagic acid on level of catalytic fragment of PKCδ. Values are expressed as mean ± SD, *indicates that groups differ significantly from DL at the level of significance *p < 0.05* using one way ANOVA followed by Tukey test. N, DL, DLT40, DLT60 and DLT80 represents normal, Dalton’s lymphoma bearing, and Dalton’s lymphoma bearing mice treated with 40, 60, 80 mg/kg body weight of ellagic acid respectively

The effect of ellagic acid was analyzed on the activity of pro-apoptotic isozyme PKCδ. The activity was measured in terms of the level of catalytic fragment (CF) by Western blot analysis. Catalytically active fragment (CF) of PKCδ was found to be down regulated significantly (*p* < 0.05) in DL mice liver as compared to normal. The dose of 60 mg and 80 mg ellagic acid increased its level significantly upto 1.64 and 1.42 folds respectively (Fig. 2).

### Ellagic acid promotes expression and activation of caspase-3

**Fig. 3:**
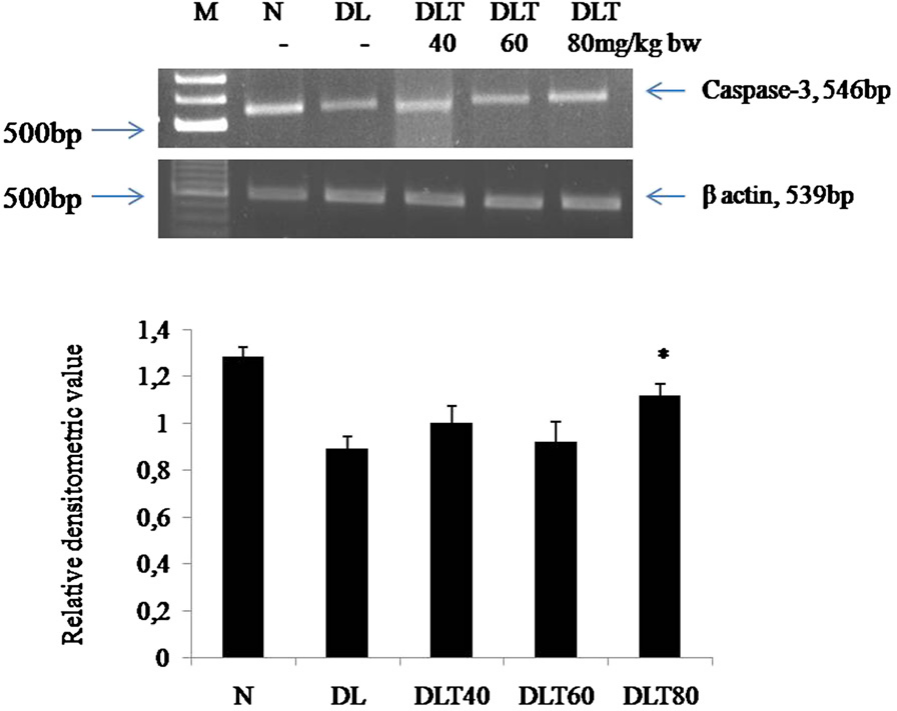
Effect of ellagic acid on gene expression of caspase-3. Values are expressed as mean ± SD, * indicates that groups differ significantly from DL at the level of significance *p < 0.05* using one way ANOVA followed by Tukey test. N, DL, DLT40, DLT60 and DLT80 represents normal, Dalton’s lymphoma bearing, and Dalton’s lymphoma bearing mice treated with 40, 60, 80 mg/kg body weight of ellagic acid respectively

**Fig. 4:**
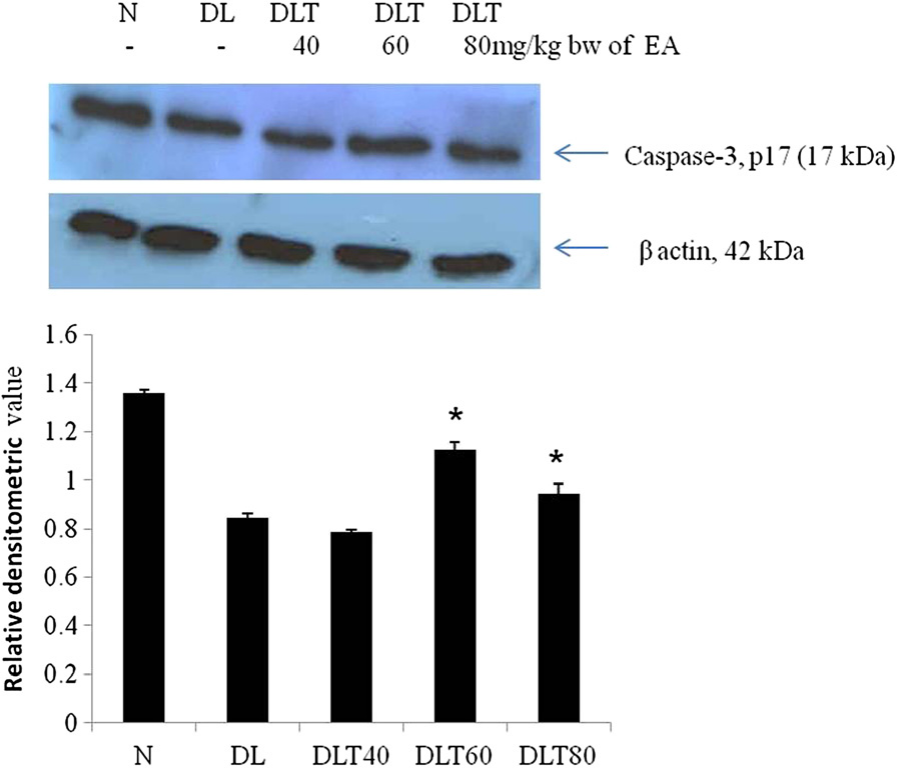
Effect of ellagic acid on activity of caspase-3 in terms of its p17 fragment by Western blot analysis in liver of mice. Values are expressed as mean ± SD, * indicates that groups differ significantly from DL at the level of significance *p < 0.05* using one way ANOVA followed by Tukey test. N, DL, DLT40, DLT60 and DLT80 represents normal, Dalton’s lymphoma bearing, and Dalton’s lymphoma bearing mice treated with 40, 60, 80 mg/kg body weight of ellagic acid respectively

The implication of ellagic acid on apoptosis was further checked by testing the apoptotic enzyme caspase-3. The mRNA expression of caspase-3 was down regulated in DL mice as compared to normal, which was significantly up regulated up to 1.3 folds with 80 mg dose of ellagic acid treatment (Fig. 3). Caspase-3 is activated only in the form of cleaved p17 peptide. Cleavage of caspace 3 was low to about 62 % in DL mouse, as tested by level of cleaved form (p17); however treatment with ellagic acid to DL mouse promotes activation of caspace 3. The dose of 60 and 80 mg/kg body weight of ellagic acid significantly increased the activation approximately up to 133 and 112 % respectively (Fig. 4).

### Ellagic acid increases the expression of atypical PKC isozymes

**Fig. 5:**
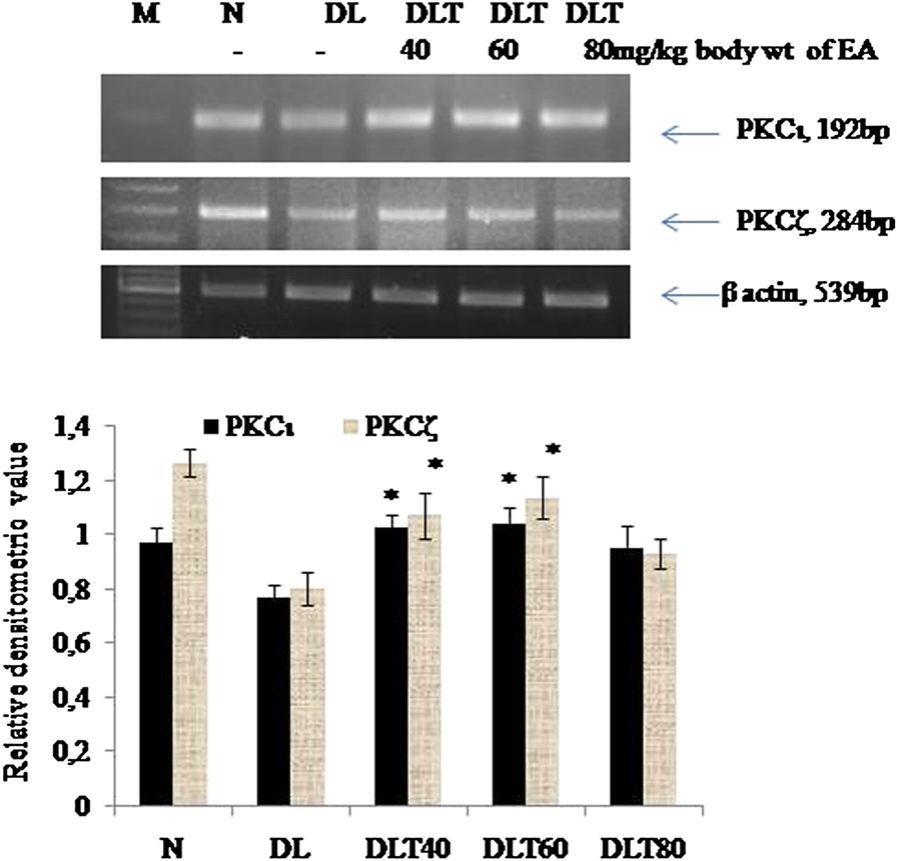
Effect of ellagic acid on gene expression of atypical PKC isozymes. Values are expressed as mean ± SD, *indicates that groups differ significantly from DL at the level of significance *p < 0.05* using one way ANOVA followed by Tukey test. N, DL, DLT40, DLT60 and DLT80 represents normal, Dalton’s lymphoma bearing, and Dalton’s lymphoma bearing mice treated with 40, 60, 80 mg/kg body weight of ellagic acid respectively

Following a similar trend of variation of novel isozymes of PKC, the expressions of atypical isozymes were also found to be decreased in DL mice liver as compare to normal; and were significantly enhanced by approximately 40 % with the doses of 40 and 60 mg ellagic acid (Fig. 5).

### Ellagic acid regulates anaerobic metabolism

**Fig. 6:**
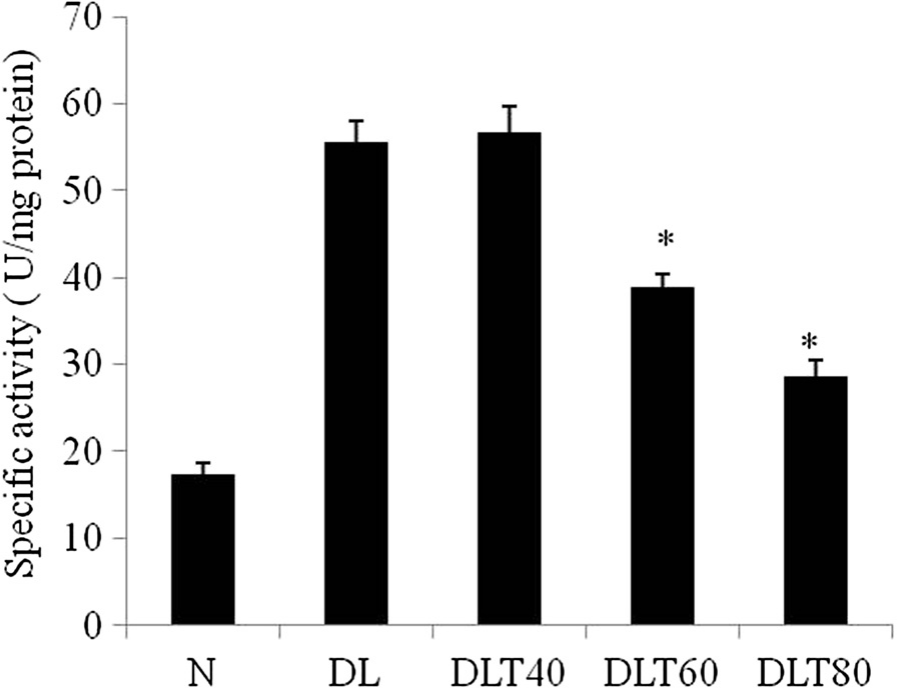
Effect of ellagic acid on total activity of LDH. Values are expressed as mean ± SD, *indicates that groups differ significantly from DL at the level of significance *p < 0.05* using one way ANOVA followed by Tukey test. N, DL, DLT40, DLT60 and DLT80 represents normal, Dalton’s lymphoma bearing, and Dalton’s lymphoma bearing mice treated with 40, 60, 80 mg/kg body weight of ellagic acid respectively

**Fig. 7:**
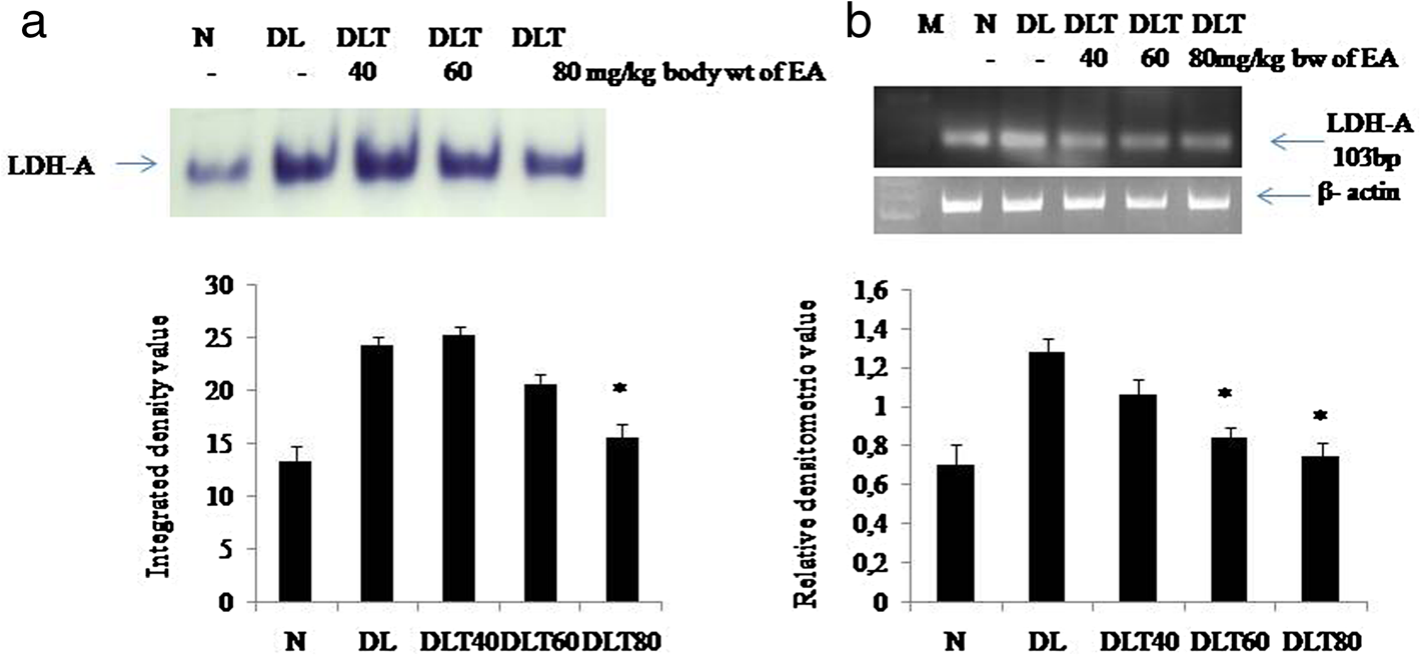
Effect of ellagic acid on activity and expression of LDH- A. **a** In gel assay showing LDH-A activity. **b** RT-PCR analysis showing mRNA expression of LDH-A. Values are expressed as mean ± SD, * indicates that groups differ significantly from DL at the level of significance *p < 0.05* using one way ANOVA followed by Tukey test. N, DL, DLT40, DLT60 and DLT80 represents normal, Dalton’s lymphoma bearing, and Dalton’s lymphoma bearing mice treated with 40, 60, 80 mg/kg body weight of ellagic acid respectively

Glycolytic metabolism was monitored in terms of total activity of LDH as well as activity of anaerobic isozymes LDH-A. Total activity of LDH was analyzed by spectrophotometric assay, which was found to be approximately 3 fold higher in DL mice liver as compared to normal. The dose of 60 and 80 mg ellagic acid was found to decrease the activity approximately by half (Fig. 6). Activity of LDH-A measured by activity gel assay, was approximately double in DL mice liver as compared to normal and dose of 80 mg ellagic acid significantly inhibited the activity (Fig. 7a). Following the similar variation pattern, the expression of LDH-A in liver of DL mice was also elevated up to about double as compared to normal and ellagic acid treatment down regulated the expression significantly in a dose dependent manner (Fig. 7b).

The dose of 80 mg ellagic acid/kg body weight was found to be optimal dose.

## Discussion

Anti carcinogenic property of ellagic acid has been established by us in DL mice by decrease in tumor size (volume of ascites fluid), decrease in viability and proliferation of ascites cells as well as increase in longevity of DL mice. Antioxidant phytochemicals are proposed to act through intracellular signaling pathways [22]. In the present study we tested the hypothesis that anti carcinogenic property of polyphenol ellagic acid could be mediated by enhancing apoptosis in cancerous tissue. We have tested here whether ellagic acid modulates novel and atypical izsozymes of PKC, as these isozymes are associated with apoptotic stimulation. The implication of ellagic acid on apoptotic process is further checked by monitoring expression and activity of caspase-3. In addition, the status of glycolytic metabolism was analyzed to assess the availability of high energy necessary for lymphoma growth.

Novel PKC is reported to be mainly involved in apoptosis. Recently, activation of PKCδ signaling is reported to induce apoptosis in hepatocellular carcinoma cells [23]. Epigenin, a plant product activates PKCδ and induces apoptosis in leukemia cells [24]. Over expression of PKCε is reported to cause apoptosis in human prostate cancer cells [25]. Recent evidences indicate that proteolytically cleaved, activated form of PKCε regulates apoptosis. PKCθ has also been reported to be involved in chemical (TCDD) induced apoptosis of lymphoblastic T cell line, L-MAT [26] and in neuroblastoma and Jurkat cells [27]. Reduced expression of PKCή has been shown in human liver cancer [28]. Our finding of decreased expression of all the isozymes of novel PKC (PKCδ, PKCε, PKCθ PKCή) in liver of lymphoma bearing mice, and their up regulation by ellagic acid treatment suggest apoptosis-inducing activity of ellagic acid. The ubiquitously expressed PKCδ is major isoform of novel PKC which has been shown to regulate apoptosis and survival in different cells [29]. PKCδ is activated under different stimuli and cleaves caspace-3 producing its active fragment i.e. p17 peptide [14]. Moreover, caspase-3 cleaves PKCδ and leads to generation of its catalytically active fragment (CF) which is implicated in the apoptotic function [30]. Decreased level of both the catalytically active fragment of PKCδ in DL mice liver and significant improvement in the level of the active fragment by ellagic acid supports cancer preventive role of ellagic acid via apoptotic stimulation by a positive feedback stimulation of the activity of both PKCδ and caspase-3.

The result suggests a novel link between the activation of PKCδ and the induction of apoptosis by ellagic acid. Our result also confirms decreased expression of caspase-3 in DL mice liver. Caspase-3 expression is reported to be variable in a variety of non-Hodgkin’s and Hodgkin’s lymphomas [31]. The mRNA and protein expressions of caspase-3 are reported to be undetectable in breast and cervical cancers and substantially low expression in ovarian cancer and in prostate tumors [32]. We demonstrate that ellagic acid induces PKCδ activity in association with proteolytic activation of caspase-3, as indicated by enhanced level of active fragment of caspase-3. Proteolytic cleavage of PKCδ by caspase-3 results in persistent activation of the PKC which might initiate a myriad of vital signalling cascades for apoptosis [33]. Thus the results support positive feedback activation loop between PKCδ and caspase-3 during apoptosis as suggested earlier [30].

Atypical PKCs regulate various cellular processes like cell proliferation, apoptosis and cell differentiation. We demonstrate decreased expression of both isoforms of atypical PKCs - PKCζ and PKCι in liver of lymphoma bearing mice and their increase after ellagic acid treatment. However, the role of PKCζ in tumorigenesis is controversial. PKCζ is reported to suppress apoptosis by directly inhibiting and interacting Bax pro apoptotic activity in lung carcinoma cells [34], as well as to induce apoptosis in ovarian cancer cells by phosphorylation of the kinase [35]. PKCζ has been reported to repress interleukin-6 promoter and impairs tumorigenesis *in vivo* [36]. Similarly, PKCι is involved in carcinogenesis of several cancers [37]. PKCι is recently reported to mediate lipid-induced apoptosis of human coronary artery endothelial cells [38]. Our result supports anti carcinogenic action of PKCζ and PKCι.

Earlier we have reported that ellagic acid regulates the classical isozymes involved in cell proliferation via NF-kB signalling [4]. In the present study, the expression of novel and atypical isozymes are found to be regulated in opposite way to classical isozymes by ellagic acid treatment, suggesting that novel and atypical PKCs are involved in apoptosis of lymphoma induced by ellagic acid.

Tumor cells exhibit profound genetic, biochemical and histological differences with respect to the original, non transformed cells types. A distinctive feature of tumor cells is the alteration of their carbohydrate metabolism to aerobic glycolytic pathway. Warburg’s effect demonstrates necessity of aerobic glycolysis or glycolytic metabolism to provide sufficient energy for maintenance of cancer growth [39]. Therefore, activation of anti survival pathway in ellagic acid treated mice should be under regulation of glycolytic metabolism.

In addition, tumor micro environment is turned acidic due to production of lactic acid during glycolytic pathway. Acidic medium stimulates disruption of adherence junctions, metastatic potential, proteinase activity and drug resistance [40]. LDH-A is one of the main isoforms of LDH which catalyses the conversion of pyruvate to lactate even under hypoxic condition of tumor microenvironment. The activity and expression of LDH-A is frequently increased in different cancers. Therefore LDH activity is considered as biochemical marker of cancer growth. Depletion of energy and growth promoting substrates due to decline in glycolytic efficiency could act as an inducer of apoptosis in the tumor cells. Decreased LDH activity, especially LDH-A activity in ellagic acid treated DL mice ensures inhibition of adequate supply of energy as well as maintenance of normal pH, resulting into poor survival of cancer cells and stimulation of apoptosis. In accordance to our result, aqueous extracts of antioxidant S. *anacardium* is reported earlier by us to down regulate anaerobic metabolism by inhibiting the activity of LDH-A in liver of lymphoma bearing mice [41]. Induced apoptosis and decreased glycolytic metabolism in DL mice, after ellagic acid treatment is correlated with its cancer preventive role. We have earlier demonstrated anti carcinogenic activity of ellagic acid in DL mice in terms of morphologic changes, as well as by increase in longevity, decrease in volume of ascites fluid accumulation in peritoneum and by decrease in cell proliferation with increased survival [4]. The overall result supports our earlier findings of cancer preventing role of ellagic acid in ascites cells of lymphoma bearing mice [42]. Thus ellagic acid prevents primary lymphoma growth in ascites fluid, as well as secondary growth in liver of DL mice by promoting apoptosis and inhibiting energy production by glycolytic metabolism.

## Conclusion

The present study demonstrates anti carcinogenic activity of ellagic acid by inducing apoptosis in liver of DL mice via novel and atypical PKCs, especially PKCδ in association with caspase-3 and also by blocking the energy metabolism.

## Abbreviations

PKC: Protein Kinase C
DL: Dalton’s lymphoma
CF: Catalytically active fragment
